# Drug and biomarker tissue levels in a randomized presurgical trial on exemestane alternative schedules

**DOI:** 10.1093/jnci/djae183

**Published:** 2024-08-07

**Authors:** Davide Serrano, Harriet Johansson, Bjørn-Erik Bertelsen, Sara Gandini, Gunnar Mellgren, Parijatham Thomas, Katherine D Crew, Nagi B Kumar, Debora Macis, Valentina Aristarco, Aliana Guerrieri-Gonzaga, Matteo Lazzeroni, Mauro D’Amico, Tania Buttiron-Webber, Irene Maria Briata, Stefano Spinaci, Viviana Galimberti, Lana A Vornik, Eduardo Vilar, Powel H Brown, Brandy M Heckman-Stoddard, Eva Szabo, Bernardo Bonanni, Andrea DeCensi

**Affiliations:** Division of Cancer Prevention and Genetics, European Institute of Oncology IRCCS, Milan, Italy; Division of Cancer Prevention and Genetics, European Institute of Oncology IRCCS, Milan, Italy; Hormone Laboratory, Department of Medical Biochemistry and Pharmacology, Haukeland University Hospital, Bergen, Norway; Division of Cancer Prevention and Genetics, European Institute of Oncology IRCCS, Milan, Italy; Hormone Laboratory, Department of Medical Biochemistry and Pharmacology, Haukeland University Hospital, Bergen, Norway; Department of Clinical Science, University of Bergen, Bergen, Norway; Department of Clinical Cancer Prevention, University of Texas, MD Anderson Cancer Center, Houston, TX, USA; Clinical Breast Cancer Prevention Program, Columbia University Irving Medical Center, New York, NY, USA; Departments Cancer Epidemiology, Genitourinary and Breast Oncology, Moffitt Cancer Center, University of South Florida, Tampa, FL, USA; Division of Cancer Prevention and Genetics, European Institute of Oncology IRCCS, Milan, Italy; Division of Cancer Prevention and Genetics, European Institute of Oncology IRCCS, Milan, Italy; Division of Cancer Prevention and Genetics, European Institute of Oncology IRCCS, Milan, Italy; Medical Oncology, Ospedali Galliera, Genoa, Italy; Medical Oncology, Ospedali Galliera, Genoa, Italy; Medical Oncology, Ospedali Galliera, Genoa, Italy; Breast Unit, Ospedale Villa Scassi ASL3, Genoa, Italy; Division of Cancer Prevention and Genetics, European Institute of Oncology IRCCS, Milan, Italy; Department of Clinical Cancer Prevention, University of Texas, MD Anderson Cancer Center, Houston, TX, USA; Department of Clinical Cancer Prevention, University of Texas, MD Anderson Cancer Center, Houston, TX, USA; Department of Clinical Cancer Prevention, University of Texas, MD Anderson Cancer Center, Houston, TX, USA; Division of Cancer Prevention, NCI, Bethesda, MD, USA; Division of Cancer Prevention, NCI, Bethesda, MD, USA; Division of Cancer Prevention and Genetics, European Institute of Oncology IRCCS, Milan, Italy; Medical Oncology, Ospedali Galliera, Genoa, Italy; Wolfson Institute of Population Health, Queen Mary University of London, UK

## Abstract

The drug’s activity at the target tissue could help to define the minimal effective dose to promote cancer preventive therapy. Here we present exemestane and sex hormone concentrations within breast tissue from a presurgical study of alternative exemestane schedules. Postmenopausal women candidates for breast surgery for estrogen receptor-positive breast cancer were randomly assigned to exemestane 25 mg once daily (QD), 25 mg 3 times/week (TIW), or 25 mg per week (QW) for 4-6 weeks before surgery. Drug and sex hormones were analyzed from homogenized frozen tissue using a QTRAP 6500+ LC-MS/MS System. Tissue drug concentrations were detectable only in the QD arm with higher concentrations in nonmalignant tissue. Estradiol was nearly suppressed in all groups in the nonmalignant tissue (QD vs TIW *P* = .364 and QD vs QW *P* = .693). In contrast, a dose-response trend was observed in cancer tissue. Based on estradiol suppression in nonmalignant tissue, lower exemestane schedules should be explored for breast cancer preventive therapy.

**Trial Registration**: Clinical Trials.gov NCT02598557 and EudraCT 2015-005063-1

Breast cancer preventive therapy has succeeded in several phase III clinical trials, using estrogen receptor modulators as well as aromatase inhibitors (1-3). However, its uptake in clinical practice is low, mainly for fear of serious adverse events or high discontinuation rate for worsening of quality of life (4,5).

To overcome this barrier, our group has extensively studied low doses of tamoxifen, and a phase III study proved that 5 mg per day of tamoxifen can substantially reduce recurrence from ductal or lobular carcinoma in situ or atypical ductal hyperplasia with negligible side effects and a carryover efficacy of up to 10 years of follow-up (6). Along this line, we have recently completed a presurgical study using alternative exemestane schedules showing a noninferior estradiol suppression of exemestane 3 times/week vs the standard daily dose in compliant participants (7).

Microenvironment stimulations have great importance in breast carcinogenesis, and estrogen levels in the mammary gland are higher compared with the plasma (8). Moreover, breast tissue estrogen concentrations are similar in postmenopausal and premenopausal women (9), with inter-individual variability related to the expression of the estrogen receptor (ER), which is higher in ER-positive cancer (10).

Here, we analyzed the concentrations of exemestane, its main metabolite 17-OH-exemestane, and sex hormones in the malignant and nonmalignant breast tissue within the above-mentioned presurgical study to further define the minimal effective dose of exemestane for therapeutic prevention.

The study design was described in detail in a recent publication (11). Briefly, this was a presurgical, 3-arm, double-blind, phase IIb trial. The main inclusion criteria were postmenopausal patients with confirmed ER-positive breast cancer candidates for surgery. Women were randomly assigned (1:1:1) to either exemestane, 25 mg, once daily, 3 times per week, or once a week for 4 to 6 weeks, time lag chosen to be able to maintain the weekly dose schedule and some flexibility for the surgeon schedule. The protocol was approved by the National Cancer Institute (NCI) Central Institutional Review Board (IRB) and the local Italian IRBs (registered at Clinical Trials.gov NCT02598557 and IEO 370 EudraCT 2015-005063-16); and the Regional Committee for Medical and Health Research Ethics in Western Norway (40213). All participants signed written informed consent. All 5 centers were encouraged to include minority races and ethnicities in the study, but the study was not powered to assess the impact of racial or ethnic or ancestry-based differences.

Snap-frozen specimens from the tumor and nonmalignant tissue were collected at surgery. The analytes were detected on Mass Spectrometer by electrospray ionization. Samples were homogenized using zirconium beads and a Tissue Lyser II, and the organic phase was transferred to glass vials and evaporated. The extracts were reconstituted in a methanol solution before analysis. The samples were analyzed using a QTRAP 6500+ LC-MS/MS System (SCIEX); positive electrospray ionization (ESI) mode was used for exemestane and 17-OH-exemestane, and negative ESI mode for sex hormones. Values of 15.5 fmol/g for estradiol, 4.5 fmol/g for estrone, 43.5 fmol/g for exemestane; 65 fmol/g for 17-OH-exemestane; and 47 fmol/g for testosterone were below the lower detection limit (12).

Serum exemestane and 17-OH-exemestane and tissue biomarkers, as median values and interquartile ranges (IQR) post-treatment, are presented by arms and type of tissue (malignant and nonmalignant). Median values and IQR of Ki-67%, PgR %, and ER % expression are presented at baseline and as the change in time by arm.

Contrasts by arms were evaluated through ANCOVA models adjusted for Body Mass Index (BMI) and age. The normal distribution of residuals from the full model was graphically checked. All *P* values were 2-sided with 5% significance level.

Out of 180 participants (155 were White non-Hispanic), we collected 94 cancer samples and 117 nonmalignant samples to analyze exemestane, 17-OH-exemestane, and sex steroids (CONSORT statement is depicted in Supplementary Figure 1, available online). All participants in this subgroup were treatment-compliant, and their age and BMI were well-balanced (data not shown).

Exemestane and 17-OH-exemestane tissue concentrations were detectable only in the once daily arm, whereas the other two arms were below the lower detection limit. Median exemestane and 17-OH-exemestane levels accumulated 4- to 5-fold in nonmalignant tissue compared with malignant tissue in the once daily arm (3807 fmol/g vs 17485 fmol/g for exemestane and 338 fmol/g vs 1343 fmol/g for 17-OH-exemestane, respectively; see Table 1).

**Table 1.** Median interquartile range of exemestane 17-OH-exemestane and sex hormones

		Exe 25 QD	Exe 25 TIW	Exe 25 QW	*P* ^a^ QD vs TIW	*P* ^a^ QD vs QW
Serum		(n = 55)	(n = 56)	(n = 60)		
	Exemestane (pmol/L)	3216.5 (2320; 4567)	513 (341; 727)	24.3 (16.6; 45.5)	<.0001	<.0001
	17-OH-exemestane (pmol/L)	1069 (644; 1657)	196 (119; 363)	7 (7; 22.3)	<.0001	<.0001
Malignant tissue		(n = 32)	(n = 31)	(n = 31)		
	Exemestane (fmol/g)	3807 (1663; 7194)	< LDL (<LDL; 291)	<LDL (<LDL; <LDL)	<.0001	<.0001
	17-OH-exemestane (fmol/g)	338 (<LDL; 1360)	<LDL (<LDL; <LDL)	<LDL (<LLOD; <LDL)	<.0001	<.0001
	Estradiol (fmol/g)	<LDL (<LDL; 52.2)	17.1 (<LLOD; 125.3)	128.8 (<LDL; 224.8)	.046	<.0001
	Estrone (fmol/g)	8.7 (<LDL; 21.7.2)	28.1 (17.8; 41.6)	138.9 (53.2; 246.7)	.017	<.0001
	Androstenedione (fmol/g)	6864 (3324; 8890)	5314 (3692; 6642)	5564 (3194; 8116)	.287	.497
	Testosterone (fmol/g)	395 (273; 567)	396 (346; 790)	453 (314; 700)	.123	.665
Nonmalignant tissue		(n = 42)	(n = 37)	(n = 40)		
	Exemestane (fmol/g)	17 485 (6791; 31 985)	435 (<LDL; 791)	<LDL (<LDL; <LDL)	<.0001	<.0001
	17-OH-exemestane (fmol/g)	1343 (262; 2758)	<LDL (<LDL; <LDL)	<LDL (<LDL; <LDL)	<.0001	<.0001
	Estradiol (fmol/g)	<LDL (<LDL; <LDL)	<LDL (<LDL; <LDL)	<LDL (<LDL; 25.5)	.364	.693
	Estrone (fmol/g)	17.35 (4.8; 33)	37.8 (21.27; 67.5)	140.5 (68.2; 233)	.012	<.0001
	Androstenedione (fmol/g)	10 251 (6541; 16 103)	12 366 (8702; 18 654)	10 580 (5403; 15 003)	.114	.490
	Testosterone (fmol/g)	436 (273; 654)	464 (341; 787)	399 (299; 570)	.181	.791

a Multivariable models adjusted for age and BMI. QD = once a day; TIW = 3 times a week; QW = once a week; LDL = lower detection limit. Exemestane 44 fmol/g, 17-OH-exemestane 65 fmol/g, estradiol 15.5, fmol/g, estrone 4.5 fmol/g, respectively.

Despite drug variability among arms, estradiol was almost completely suppressed in all arms in nonmalignant tissue, showing the median and the inter-range quartile below detectable levels in the once daily and 3 times per week arms, and only the upper quartile reached detectable levels in the once a week arm, with no differences in once daily vs 3 times per week and once daily vs once a week (*P* = .364 and *P* = .693, respectively). Conversely, a dose-response trend was observed in cancer tissue, with the estradiol level being below the lower detection limit (<LDL) (<LDL, 52.2 fmol/g) on once daily, 17.1 (<LDL, 125.3) on 3 times per week, and 128 (<LDL, 224.8) on once a week (*P* = .046 once daily vs 3 times per week arms). Figure 1 shows the percentage of participants below the lower detection limit for estradiol. Interestingly, in malignant tissue, there was no statistical difference (*P* = .056) between once daily and 3 times per week in the percentage of women who reached estradiol suppression. However, the once a week arm had more women who did not reach estradiol LDL, although the median level was similar among arms (Supplementary Table 1, available online). Estrone showed a clear dose-response trend among arms, whereas no differences were observed for testosterone and androstenedione for both malignant and nonmalignant tissue in all arms. A comparison between tissue distribution for both hormones is depicted in Supplementary Table 2 (available online), with no clear evidence of a differential distribution for estrone and a strong difference for androstenedione. The Ki-67 and hormones receptor expression change in cancer tissue was previously shown (7). Here we report the data for those patients for which tissue samples were available. An absolute change (IQR) from baseline of Ki-67 of -8 (-10, -3), -6 (-11, -2), -4 (-8, -1) was observed in once daily, 3 times per week, and once a week, respectively (Supplementary Table 3, available online). The Ki-67 reduction in this subgroup was similar to the whole study population (7). Similarly, progesterone receptors were reduced by the treatment, whereas no changes were observed for the estrogen receptor. For adverse events, there were no significant differences among the 3 arms (data not shown), as reported previously (7).

**Figure 1.**
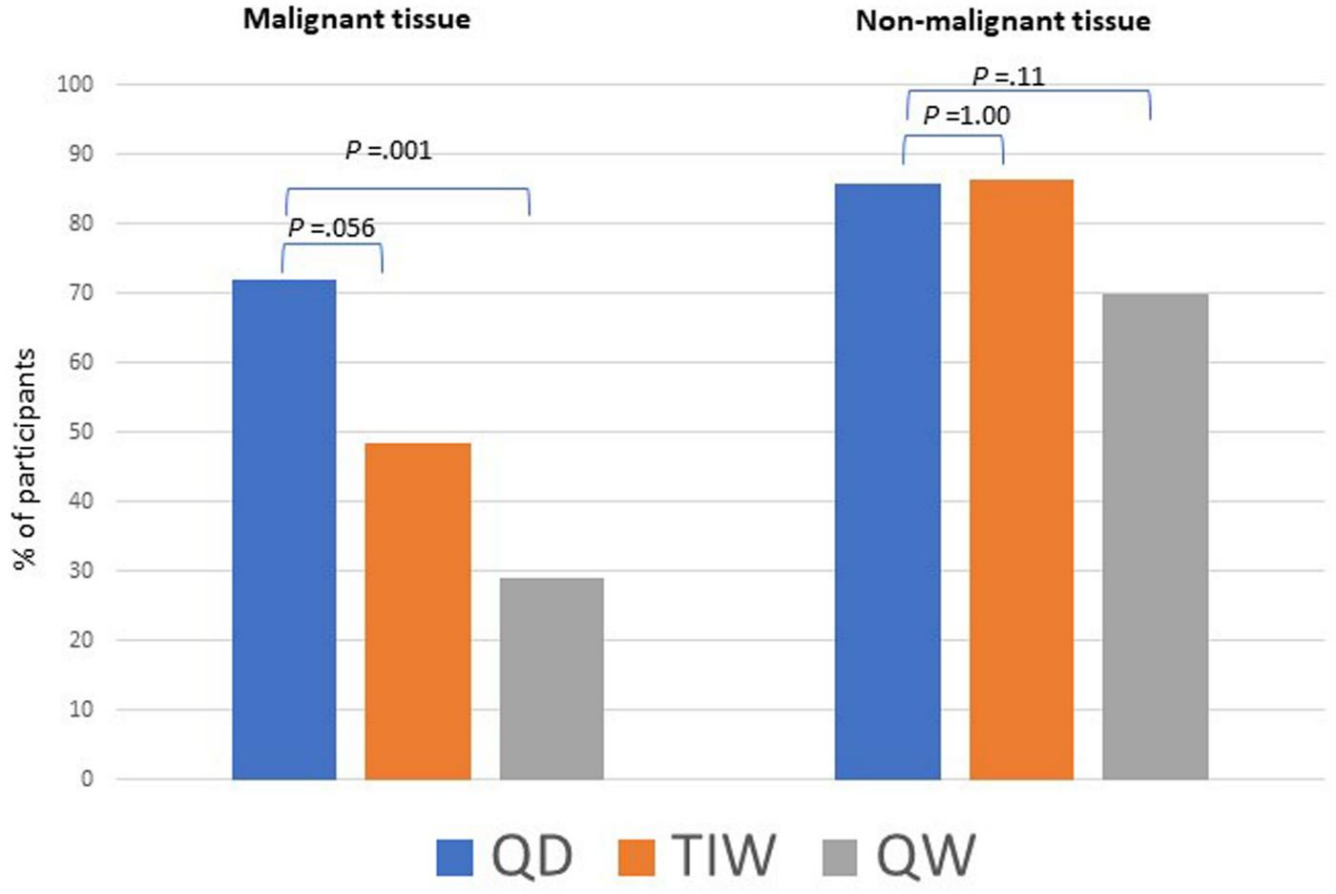
Percentage of participants with estradiol suppression below the detection limit (the analysis was done using χ^2^ test). QD = once a day; QW = once a week; TIW = 3 times a week.

Circulating estradiol is a breast cancer risk factor in postmenopausal women (13), and its suppression by anastrozole predicts efficacy in preventing breast cancer (14). Estradiol can be synthesized within normal breast and cancer tissue through aromatase and sulfate pathways (9). Seeking the minimal effective dose of exemestane, we have shown that 25 mg 3 times/week was noninferior to the standard dose in reducing circulating estradiol (7). Here we investigate the effects of the different schedules within the breast tissue. Exemestane has a relatively short half-life (27 hours), and in the tissue it was detectable only in the daily dose arm, with much higher concentrations in nonmalignant compared to malignant tissue (Table 1). Tamoxifen also showed a trend to accumulate in normal compared with malignant tissue (15), but exemestane at the standard dose shows similar levels between serum and tissue, whereas tamoxifen level was much lower in the serum compared with the tissue.

Importantly, irrespective of drug concentrations, estradiol levels in nonmalignant tissue were very low in all arms, further supporting the rationale for assessing lower doses for breast cancer prevention. Conversely, a dose-response trend was seen in the cancer tissue, possibly due to the different drug distribution within the gland (7).

The weekly dose shows barely detectable levels of estradiol in nonmalignant tissue, whereas the suppression at the serum level was different from the other 2 schedules. This difference between tissue and serum may contribute to maintaining cancer prevention activity with potentially minor systemic symptoms of estrogen deprivation, a question to be addressed in future studies.

This study has some limitations, including the lack of samples from all participants and the tissue estradiol at baseline due to limited material from the biopsies.

These results have to be taken as exploratory but support the notion that exemestane can be used at lower doses. We have launched a randomized phase II study comparing tamoxifen vs exemestane both given every other day in postmenopausal women with intraepithelial neoplasia or at higher risk for breast cancer to evaluate menopausal symptoms and biomarker modulation. Considering the estradiol suppression in nonmalignant tissue, a weekly dose might even be evaluated for healthy women at increased risk for breast cancer.

## Supplementary Material

djae183_Supplementary_Data

## Data Availability

The data underlying this article will be shared on reasonable request to the National Cancer Institute (NCI) at the following website: https://cdas.cancer.gov/learn/eppt/browse/.
