# Correction to: Drug and biomarker tissue levels in a randomized presurgical trial on exemestane alternative schedules

**DOI:** 10.1093/jnci/djaf018

**Published:** 2025-01-28

This is a correction to:

Davide Serrano, Harriet Johansson, Bjørn-Erik Bertelsen, Sara Gandini, Gunnar Mellgren, Parijatham Thomas, Katherine D Crew, Nagi B Kumar, Debora Macis, Valentina Aristarco, Aliana Guerrieri-Gonzaga, Matteo Lazzeroni, Mauro D’Amico, Tania Buttiron-Webber, Irene Maria Briata, Stefano Spinaci, Viviana Galimberti, Lana A Vornik, Eduardo Vilar, Powel H Brown, Brandy M Heckman-Stoddard, Eva Szabo, Bernardo Bonanni, Andrea DeCensi, Drug and biomarker tissue levels in a randomized presurgical trial on exemestane alternative schedules, *JNCI: Journal of the National Cancer Institute*, Volume 116, Issue 12, December 2024, Pages 1979–1982, https://doi.org/10.1093/jnci/djae183

In the originally published version of this manuscript, author, Eduardo Vilar’s, name was misspelled.

This error has been corrected.

