# Supplementary material for: Drug and biomarker tissue levels in a randomized presurgical trial on exemestane alternative schedules
Source: J Natl Cancer Inst. 2024 Aug 7;116(12):1979–82. doi: 10.1093/jnci/djae183 (PMC11630545; doi:10.1093/jnci/djae183)
Supplement: djae183_Supplementary_Data [file djae183_supplementary_data.zip › djae183_Supplementary_Data/Supplementary Tables.pdf]

Supplementary Table 1 Median and interquartile ranges of estradiol (fmol/g) in non-malignant tissue for subjects with detectable levels

**ARMS**	N	Median Estradiol	Quartile Low	Quartile High
**QD**	6	45.25	30.9	86.5
**TIW**	5	34.3	29.1	56.5
**QW**	12	43.15	29.25	63.7

LDL 15.5 fmol/g

Supplementary Table 2 Median and interquartile ranges of estrone and androstenedione (fmol/g) in malignant and non-malignant tissue

**Estrone**		N	Median	Quartile Low	Quartile High	P-value
**QD**	Malignant	32	8.7	4.5	21.7	0.1976
	Nonmalignant	42	17.4	4.8	33.0	
**TIW**	Malignant	30	25.5	17.8	39.1	0.0519
	Nonmalignant	37	37.8	21.7	67.5	
**QW**	Malignant	31	138.9	53.2	246.7	0.7283
	Nonmalignant	39	145.5	70.0	233.3	
**Androstenedione**						
**QD**	Malignant	32	6864.1	3323.6	8890.35	0.001
	Nonmalignant	42	10251.05	6541.4	16102.9	
**TIW**	Malignant	30	5310.5	3692.3	6642	<.0001
	adjacent	37	12366.4	8702.7	18654.2	
**QW**	Malignant	31	5564.4	3194.4	8115.9	0.0032
	Nonmalignant	39	10733.6	5388.1	15321.6	

Supplementary Table 3. Median (IQR) Ki-67 and hormone receptor changes in the subgroup with cancer tissue samples

**Malignant -Tissue**	**Exe 25 QD (n=32)**		**Exe 25 TIW (n=30)**		**Exe 25 QW (n=31)**	
**IHC Expression**	**Baseline**	**Change at surgery**	**Baseline**	**Change at surgery**	**Baseline**	**Change at surgery**
**Ki67 %**	13 (9; 17)	-8 (-10; -3)	14 (7; 21)	-6 (-11; -2)	13 (8; 22)	-4 (-8; -1)
**PgR % expression**	80 (30; 95)	-30 (-60; -5)	55 (3; 99)	-9 (-35; 0)	70 (20; 95)	-15 (-25; 0)
**ER % expression**	99 (95; 99)	0 (-4; 0)	99 (95; 99)	0 (-4; 4)	99 (95; 99)	0 (0; 0)
