# Supplementary figures and images for: Drug and biomarker tissue levels in a randomized presurgical trial on exemestane alternative schedules
Source: J Natl Cancer Inst. 2024 Aug 7;116(12):1979–82. doi: 10.1093/jnci/djae183 (PMC11630545; doi:10.1093/jnci/djae183)

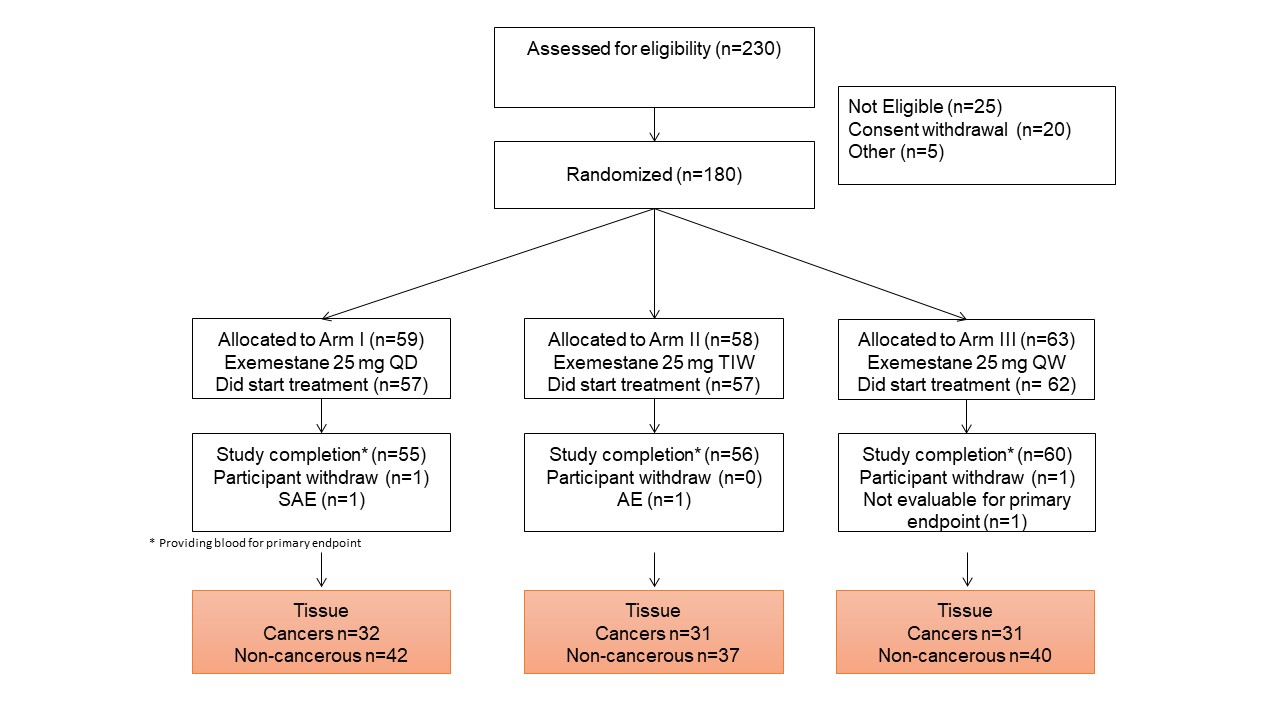

Supplement: djae183_Supplementary_Data [file djae183_supplementary_data.zip › djae183_Supplementary_Data/Supplementary figure 1.JPG]
